# Therapeutic potential of targeting *HSPA5* through dual regulation of two candidate prognostic biomarkers *ANXA1* and *PSAT1* in osteosarcoma

**DOI:** 10.18632/aging.202258

**Published:** 2020-12-03

**Authors:** Xiaojun Tang, Lingli Luo, Yukun Li, Hailong Wu, Qing Hu, Haiyan Yue, Xiao He, Juan Zou, Shaoxiong Min

**Affiliations:** 1 Department of Spinal Surgery, Orthopaedic Medical Center, Zhujiang Hospital, Southern Medical University, Guangzhou 510280, Guangdong Province, China; 2 Department of Spinal Surgery, The Second Affiliated Hospital, University of South China, Hengyang 421001, Hunan Province, China; 3 Medical College, Hunan Polytechnic of Environment and Biology, Hengyang 421005, Hunan Province, China; 4 Hunan Province Key Laboratory of Tumor Cellular and Molecular Pathology, Cancer Research Institute, University of South China, Hengyang 421001, Hunan Province, China; 5 Department of Spinal Surgery, Peking University Shenzhen Hospital, Shenzhen 518036, Guangdong Province, China; 6 Department of Pathology, People’s Hospital of Hunan Province, Changsha 410005, Hunan Province, China; 7 Department of Pathology, The Central Hospital of Shaoyang, Shaoyang 422000, Hunan Province, China; 8 Department of Breast Surgery, Hunan Provincial Tumor Hospital, Changsha 410005, Hunan Province, China

**Keywords:** osteosarcoma, prognosis, *HSPA5*, *ANXA1*, *PSAT1*

## Abstract

Osteosarcoma is the most common primary malignant bone tumor that mostly affects young people’s health. The prognosis of patients with unresectable or recurrent osteosarcoma still remains dismal. Based on gene integration analysis from GEO and TARGET databases by R language, the differentially expressed genes of osteosarcoma patients were identified. Biological molecular function analysis indicated that these genes were importantly enriched in the process of cell adhesion molecule binding. Gene significance highly-related to clinical traits of osteosarcoma was found by weighted gene co-expression network analysis. Additionally, receiver operating characteristic curve analysis was conducted to find prognostic markers in LASSO Cox regression model. Two candidate biomarkers, *ANXA1* and *PSAT1*, for the prognosis of osteosarcoma were detected separately on the basis of WGCNA and LASSO model. Of note, their expression profiles were interrelated with an important therapeutic target *HSPA5*. *In vitro* pharmaceutical experiments were performed to explore the biological role and prognostic benefit of candidates. Suppression of *HSPA5* effectively upregulated *ANXA1* and inhibited *PSAT1*, resulting in osteosarcoma cell proliferation arrest and apoptosis. These findings suggest that *HSPA5* serves as a core molecule for osteosarcoma therapy due to its bidirectional regulation of candidate prognostic biomarkers *ANXA1* and *PSAT1*.

## INTRODUCTION

Osteosarcoma (OS) is a highly aggressive bone cancer for which treatment has remained essentially unchanged over the past 30 years [1]. It is frequently found in the long bones of children and adolescents. Combination of surgery and chemotherapy can dramatically improve the outcome of patients with OS where the 5-year survival rate has reached 60% ~ 70% over the last few years [2]. Unfortunately, the majority of patients have poor responses to chemotherapy and suffer unpredictable recurrences *in situ* or lung metastasis [3]. The molecular basis of OS progression remains poorly understood. Due to no effective therapeutic targets and diagnostic markers for OS, the improvement of prognosis is strongly limited. Therefore, it is necessary to find functional prognostic biomarkers for OS.

Glucose-regulated protein 78 (GRP78) is a stress-inducible chaperone that is encoded by heat shock protein family A member 5 (*HSPA5*). It has been strongly involved in poor prognosis, such as drug resistance and lung metastasis in patients with OS [4, 5]. Studies showed that activation of unfolded protein response (UPR) by GRP78 protected OS cells from cisplatin-induced apoptosis through NF-κB pathway [6, 7]. Inhibiting GRP78 could significantly enhance the expression of tumor suppressor ATF4 via stabilizing apoptotic inducer CHOP protein [8, 9]. *HSPA5* in human OS has been recognized as a key diagnostic and prognostic biomarker [10]. Thus, the precise molecular mechanisms underlying the progression of OS driven by *HSPA5* will improve the clinical outcomes.

With the help of bioinformatics methods and recent advances in genome-wide studies, we integrated the available data of clinical samples to explore candidate prognostic biomarkers and their biological functions. We finally determined annexin A1 (*ANXA1*) and phosphoserine aminotransferase 1 (*PSAT1*) as our genes of interest. *ANXA1* serves as a tumor suppressor expressing very little in the proliferating basal OS cells which is negatively regulated by the oncogene serine/arginine-rich splicing factor 3 (*SRSF3*) [11]. In contrast, *PSAT1* is a representative metabolism-associated gene implicated in serine biosynthetic pathway (SSP), and its oncogenic activation is maintained in Ewing sarcoma [12]. Our present study highlights the critical role of *HSPA5* in the bidirectional regulation of the two prognostic indicators *ANXA1* and *PSAT1*. Targeting *HSPA5* seems to be a productive therapeutic strategy to ensure the good prognosis of OS patients.

## RESULTS

### Differentially expressed genes (DEGs) identification in OS

**Figure 1:**
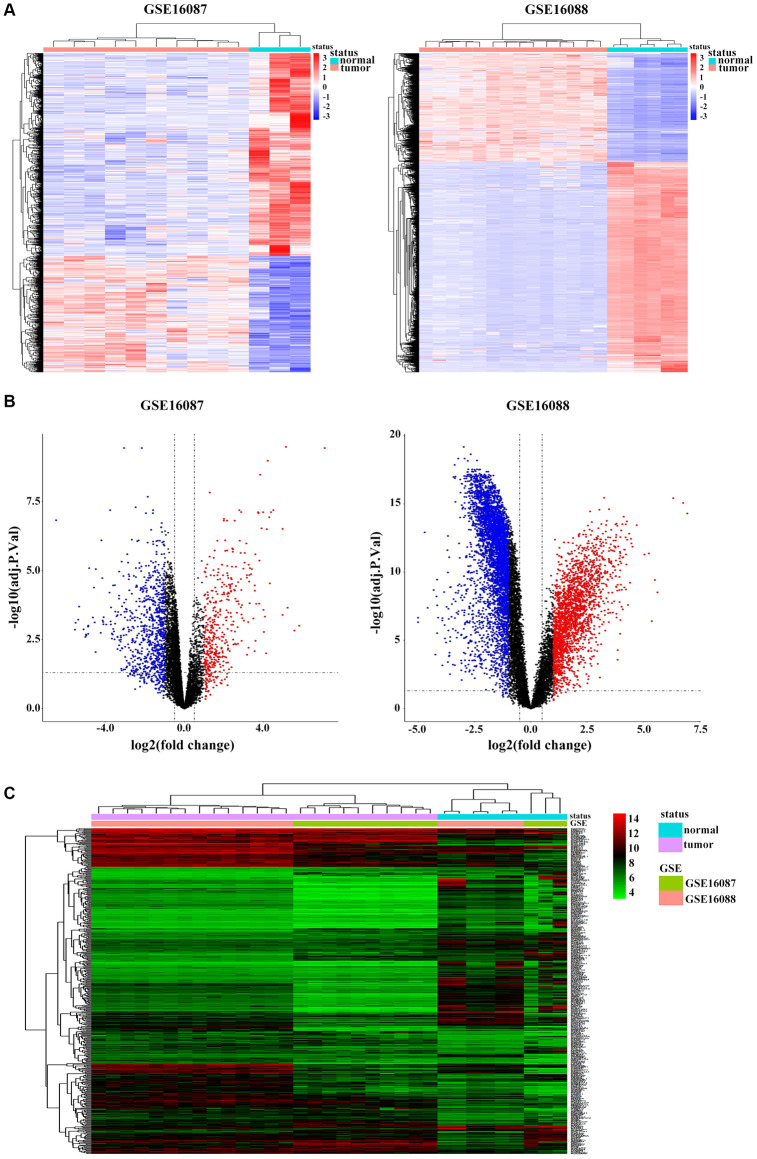
**Identification of DEGs in OS.** (**A**) Heatmap shows differential expression profiles in normal tissues and tumor tissues from the GSE16087 and GSE16088 datasets. DEGs were defined with |log_2_FC| > 1 and adjusted *P*-value < 0.05. (**B**) Genome-wide gene expression profiles of OS tumor and normal tissues from two GSE datasets were shown with volcano plots. Black symbols represent normally expressed genes. Red and blue symbols represent the aberrantly expressed genes with |log_2_FC| > 1 and adjusted *P*-value < 0.05. (**C**) Hierarchical clustering analysis of differential expression profiles of 515 common DEGs in OS tumor and normal tissues from the two GSE datasets. Blue and red blockages respectively indicate the expression level of genes lower or higher than the median expression value across all samples.

Two datasets of OS were downloaded from GEO database. After gene expression data integration of GSE16087, we identified 1200 DEGs with 762 genes downregulated and 438 genes upregulated in *in-vivo* OS canine model samples compared with those in normal control tissues. From GSE16088 dataset, we recognized 6305 DEGs, of which 2153 genes 4152 genes were downregulated and were upregulated in OS patients (Figure 1A). The volcano plots were illustrated in Figure 1B. Based on the cut-off criteria, we obtained 515 overlapping DEGs from these two GSE datasets (Supplementary Figure 1A), and the cluster heatmap is shown in Figure 1C.

### Functional characterization of OS specific genes

**Figure 2:**
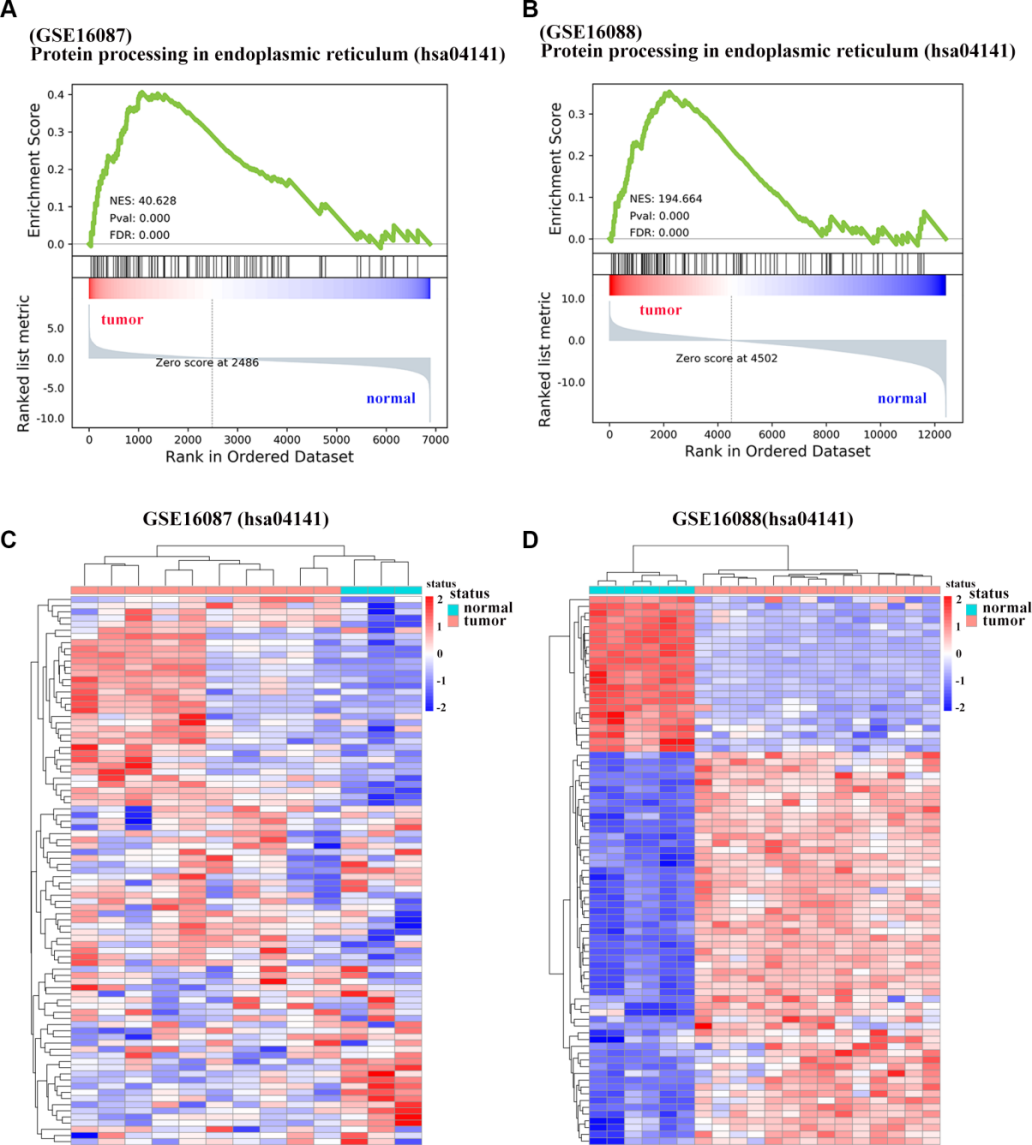
**Significantly enriched pathways of DEGs in OS.** (**A**, **B**) Representative GSEA of KEGG pathway gene set has04141 among DEGs from GSE16087 and GSE16088 datasets. (**C**, **D**) Heatmap analysis of shared DEGs enriched in KEGG pathway hsa04141 from the two GSE datasets. DEGs were defined with |log_2_FC| > 1 and adjusted *P*-value < 0.05.

**Figure 3:**
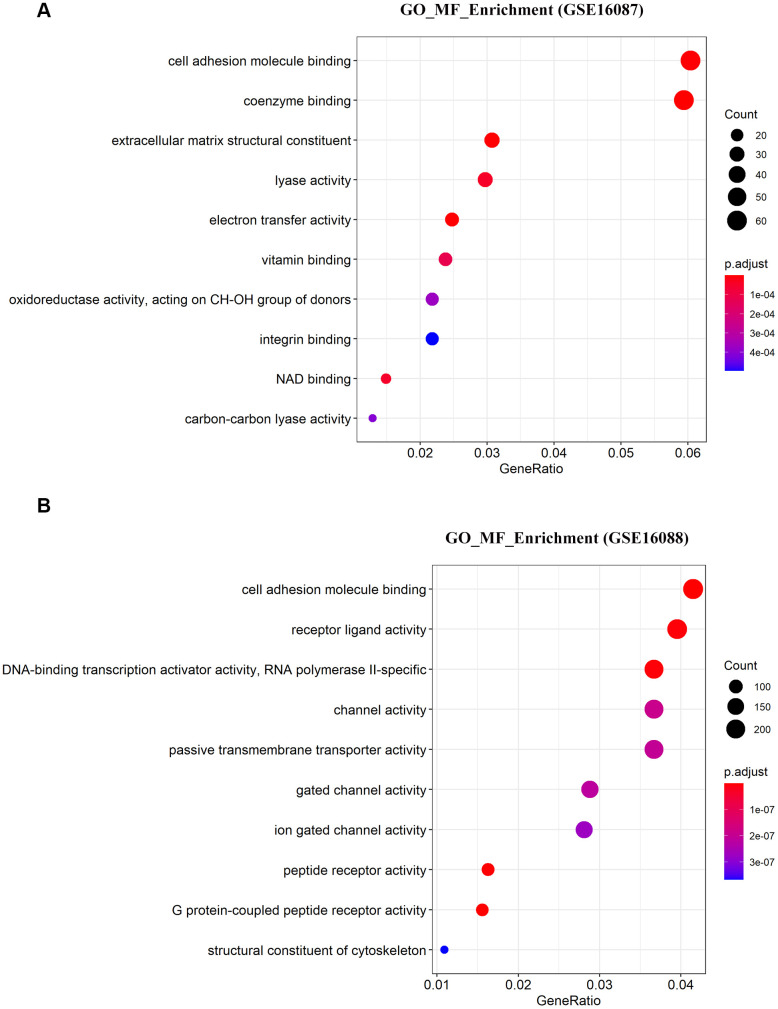
**Significantly enriched molecular function of DEGs in OS.** (**A**, **B**) The top 10 significantly enriched molecular function terms of DEGs in OS are shown using GSEA.

To determine the enriched gene sets among the DEGs, KEGG pathway analysis and GO functional analysis were run on the GSEAPY wrapper. KEGG pathway enrichment analysis exhibited that the DEGs in GSE16087 were mainly associated with protein processing in endoplasmic reticulum (ER), PI3K-Akt signaling pathway, MAPK signaling pathway and pathways in cancer. Proteasome, oxidative phosphorylation, DNA replication as well as the protein processing in ER were significantly enriched in GSE16088 (Figure 2A, 2B; Supplementary Figure 1B, 1C). The core group protein processing in ER (hsa04141) with 89 shared DEGs, were visualized by hierarchical clustering (Figure 2C, 2D). GO molecular function prediction demonstrated that the DEGs in both two datasets were primarily enriched in the process of cell adhesion molecule binding (Figure 3A, 3B). Furthermore, we gained 38 overlapping DEGs in the cell adhesion molecule binding term from the above mentioned two GSE datasets, and the biological network of GO categories was achieved by BiNGO analysis. Results showed that protein binding was statistically overrepresented in this set of genes (Supplementary Figure 2A). The PPI network complex of the overlapping DEGs was performed in the online database STRING to discover protein functions (Supplementary Figure 2B). We focused on *HSPA5*, an important ER molecular chaperone in the network. Given the vital functions of *HSPA5*-encoding chaperone protein GRP78 implicated in the poor prognosis of OS and the lack knowledge of its relationship with tumor suppressor *ANXA1* [9, 11], it prompted us to address the underlying relevance between these two important biomarkers in OS. Moreover, the shared DEGs in the GO term of interest from the two GSE datasets were conducted for GO analysis. In consistence with the biological and PPI networks, both of the *HSPA5* and *ANXA1* were enriched in the process of cadherin binding and cell adhesion molecule binding (Supplementary Figure 2C), suggesting their potentially close correlation in molecular level.

### Weighted gene co-expression network analysis (WGCNA) of *ANXA1*-centered module

**Figure 4:**
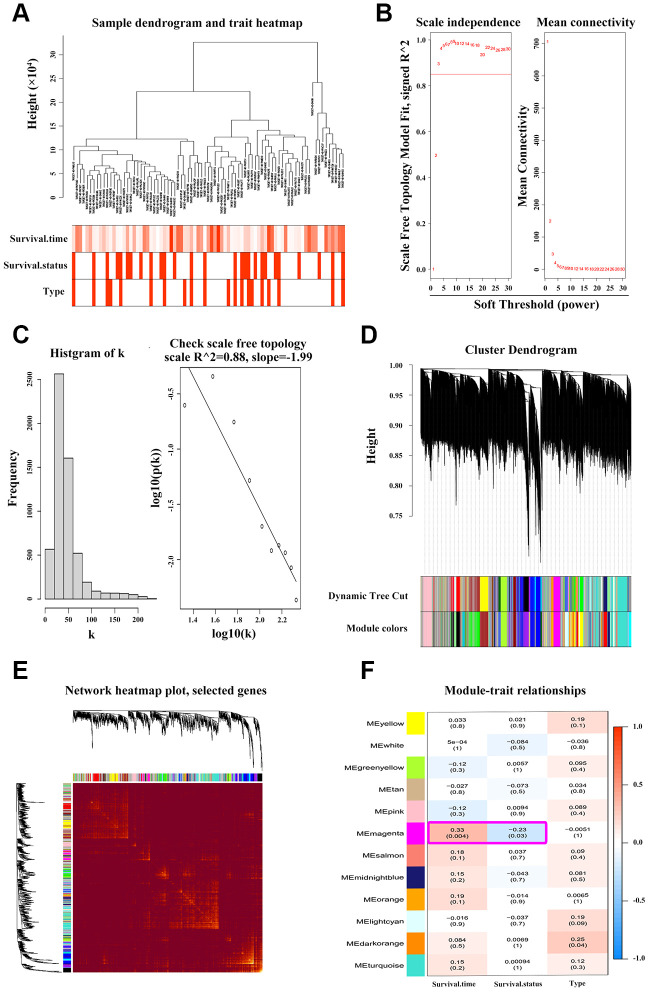
**WGCNA discoveries the key ANXA1-entered module.** (**A**) Hierarchical clustering dendrogram of tumor samples from TARGET_OS with the indicated clinical traits. (**B**–**C**) Scale independence and mean connectivity analyses of OS samples. Soft-thresholding power β = 3 fits the scale-free topology. (**D**) Clustering tree of genes with divergence. The branches of tree represent different modules. (**E**) Heatmap of TOM among 1000 genes which were selected randomly in WGCNA. Dark/light color corresponds to the degree of overlap. (**F**) Relevance of module eigengenes with traits. Based on the correlation and *P*-value in each cell, the magenta module containing *ANXA1* is selected.

**Figure 5:**
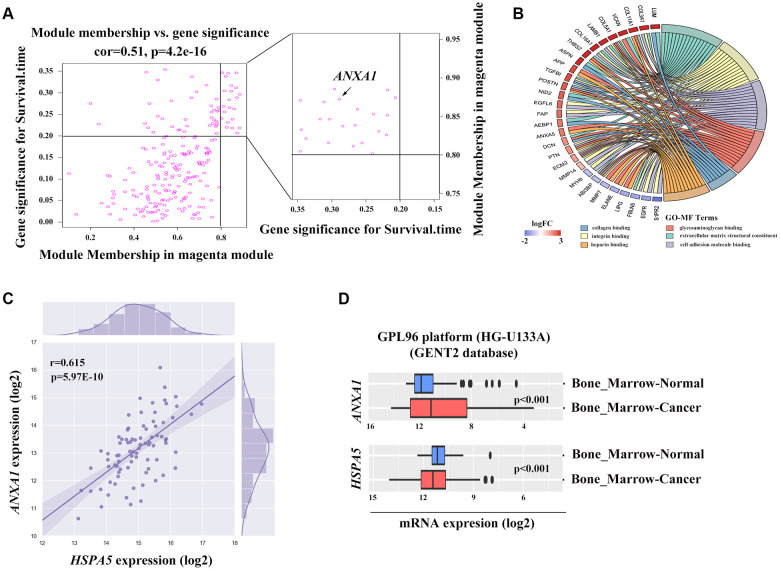
**The potential association between *ANXA1* and *HSPA5*.** (**A**) Genes involved in magenta module shown in scatter plot. Cut-off of module membership = 0.8 and gene significances for survival time = 0.2 as the criteria for the hub genes where *ANXA1* included. (**B**) Gene ontology analysis of genes linked to *ANXA1* expression based on the topological overlap. (**C**) The positive correlation between *ANXA1* and *HSPA5* mRNA expression levels (log2) in TARGET_OS dataset. Statistical *P*-value was obtained by Pearson correlation analysis. (**D**) *ANXA1* and *HSPA5* expression levels in bone marrow cancer and normal tissues through GENT2 online analysis.

To find the clusters/modules containing highly correlated genes with similar expression pattern, we studied the OS samples with clinical traits using WGCNA [13–15]. Gene expression profiles were obtained from TARGET database. In all, 81 OS samples with survival data and metastatic status were included in WGCNA for subsequent analysis (Figure 4A). Here, β = 3 and R^2^ = 0.85 were recognized as the soft-thresholding for scale-free network (Figure 4B, 4C). The grey module was excluded using merged dynamic tree cut. As a result, we finally identified 31 gene co-expression modules (Figure 4D). The heatmap plotted the topological overlap matrix (TOM) among 1000 genes randomly selected, indicating there were independent variables between the models (Figure 4E). Next, we found a key magenta module containing *ANXA1* which was highly correlated with OS prognosis (R^2^ = 0.33, *P* = 0.004 with survival time; R^2^ = -0.23, *P* = 0.03 with survival status; Figure 4F). The scatter plots of the correlation between module membership (MM) and gene significance (GS) in the magenta module with survival time of OS patients (correlation = 0.51, *P* = 4.2e−16) further illustrated that *ANXA1* was one of the genes within the limits of MM > 0.8 and GS > 0.2 (Figure 5A), suggesting that *ANXA1* was a hub gene in the magenta module. Importantly, GO analysis further indicated that the co-occurrence genes associated with *ANXA1* based on the topological overlap in WGCNA were mainly enriched in binding term, including cell adhesion molecule binding, which was in line with the results in GSE datasets (Figure 5B).

### High expression of *HSPA5* with *ANXA1* repression are associated with the progression and poor prognosis of OS

**Figure 6:**
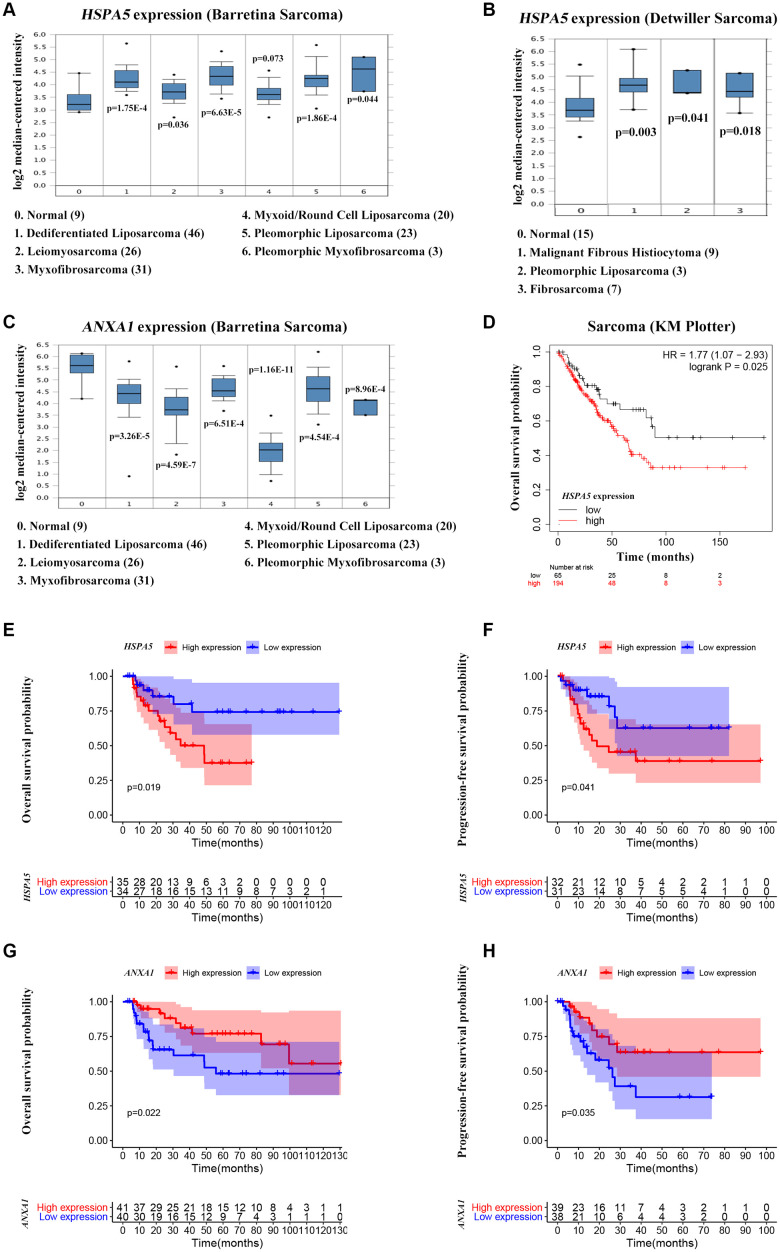
**High *ANXA1* expression and low *HSPA5* expression are involved in good outcomes of OS patients.** (**A**–**C**) Box plots showing differential mRNA levels of *ANXA1* and *HSPA5* between sarcoma and normal tissues from ONCOMINE database. (**D**) The correlation of *HSAP5* expression with the overall survival of sarcoma patients shown in Kaplan-Meier curve from KM plotter database. (**E**, **F**) The correlation of *HSAP5* expression with the overall survival (69 cases) and progression free survival (63 cases) of OS patients shown in Kaplan-Meier curves from TARGET_OS dataset. (**G**, **H**) Kaplan-Meier analyses of overall survival (in 81 cases) and progression free survival (in 77 cases) were conducted to show OS patients with lower *ANXA1* mRNA level live shorter from TARGET_OS dataset.

*ANXA1* and *HSPA5* were speculated to be intersect at multiple levels on the basis of above bioinformatic results. To address this possibility, we first assessed the correlation of expression levels between these two genes in OS patients in TARGET database. Unfavorably, the mRNA level of stress adaptive gene *HSPA5* was statistically positively associated with the tumor suppressor gene *ANXA1*(Figure 5C). To further investigate the roles of *ANXA1* and *HSPA5* in the progression of OS, we analyzed their expressions in GENT2 database. Intriguingly, *HSPA5* aberrantly expressed in bone-related cancer tissues compared with the normal tissues, whereas *ANXA1* expression appeared to be downregulated in cancer (Figure 5D). The similar results were observed in sarcoma samples from ONCOMINE database (Figure 6A–6C), and online survival analysis revealed that abnormal *HSPA5* expression resulted in significantly shorter survival time compared to that maintained in lower level (HR = 1.77, *P* = 0.025; Figure 6D). For further clinical survival evaluation, the expression level of *HSPA5* and *ANXA1* were introduced into the TARGET_OS clinical file. Kaplan-Meier survival analysis revealed that OS patients with high level of *HSPA5* mRNA showed a remarkably lower overall survival rate and progression free survival rate. In contrast, and patients with higher *ANXA1* mRNA level were inclined to live longer (Figure 6E–6H). Similar results were observed in GSE16091 dataset (Supplementary Figure 3A, 3B).

### Identification of key genes for prognosis of OS

**Figure 7:**
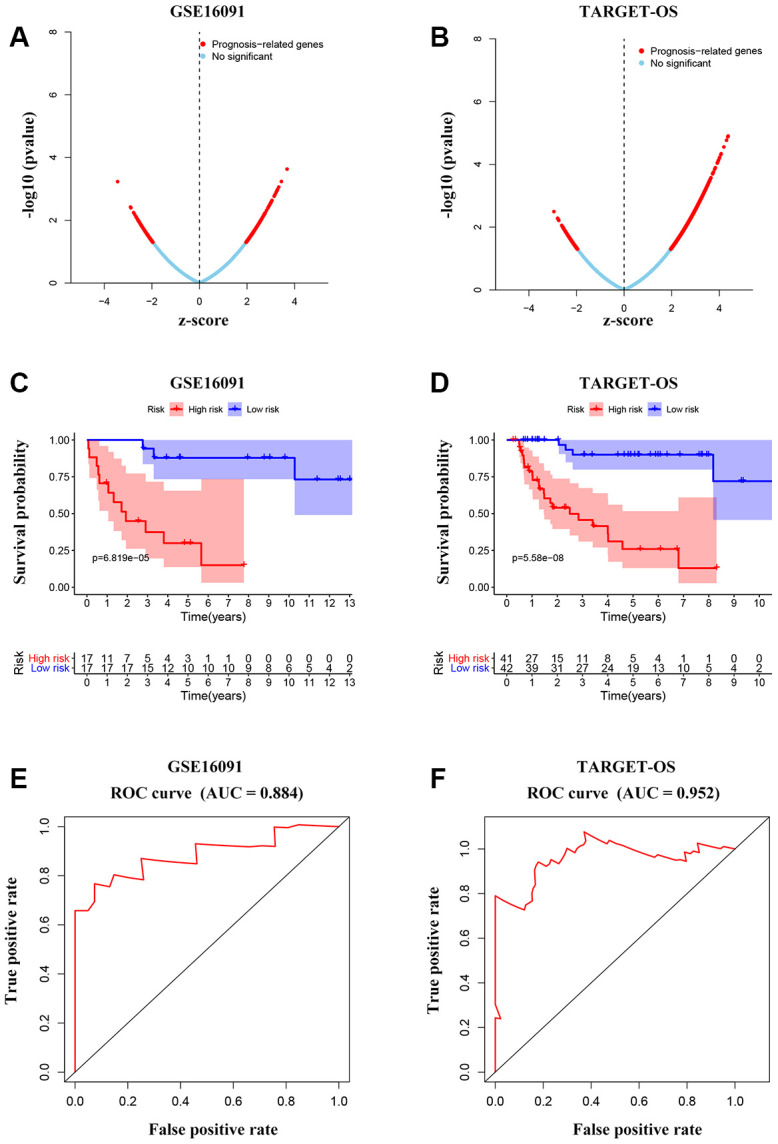
**Integrated gene signature for OS prognosis prediction.** (**A**, **B**) Univariable Cox regression analysis to find prognosis-associated genes from GSE16091 and TARGET_OS. (**C**, **D**) Kaplan-Meier curves showing the overall survival according to risk score of the set of prognosis-related signature OS patients in the two datasets. (**E**, **F**) ROC analysis of the cluster of prognostic biomarkers for OS in TARGET database and GSE16091 dataset.

**Figure 8:**
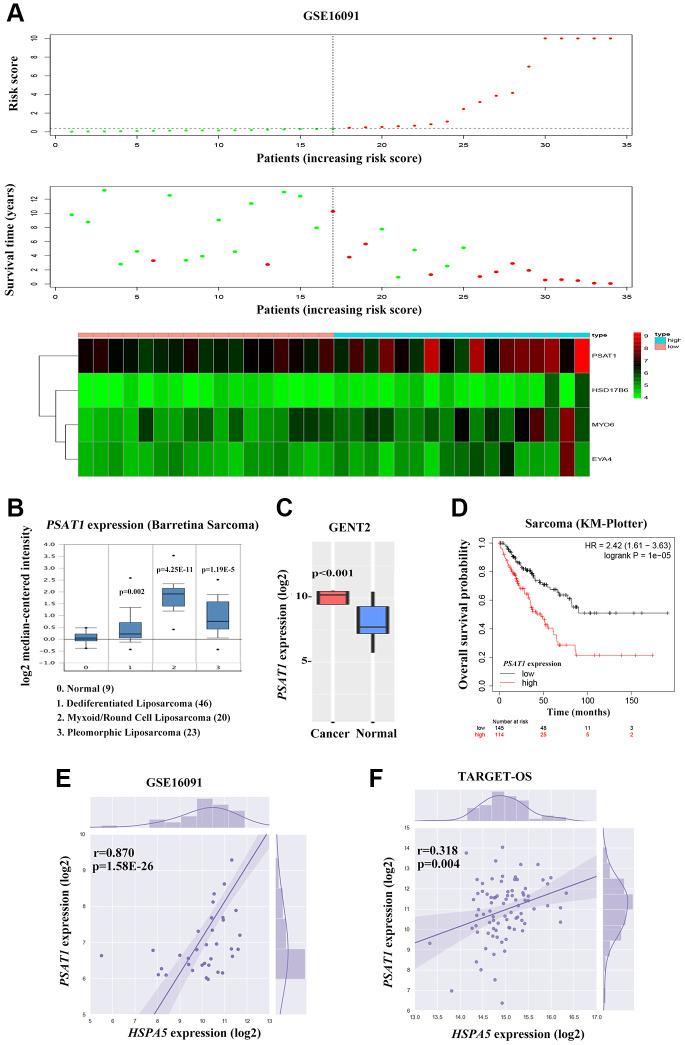
**Cross-talk between *HSPA5* and the leading OS prognosis-associated predictor *PSAT1*.** (**A**) The distribution of risk scores is shown for the cohorts from the dataset GSE16091 (upper panel). The alive are shown in green, while the dead are shown in red. And the heatmap of expression profiles of prognostic gene signature. *PSAT1* ranks first where high expression indicates poor prognosis of OS patients. (**B**) Box plot to show differential mRNA level of *PSAT1* in sarcoma tumor and normal tissues from ONCOMINE database. (**C**) *PSAT1* expression levels in bone cancer and normal tissues through GENT2 online analysis. (**D**) Kaplan-Meier curves to show the overall survival of sarcoma patients about *PSAT1* gene expression from online KM plotter database. (**E**, **F**) The positive correlation between *PSAT1* and *HSPA5* mRNA expression levels (log2) in GSE16091 and TARGET_OS datasets. Statistical *P*-value was obtained by Pearson correlation analysis.

To further investigate the candidate genes for the prognosis of OS, we separately performed the univariable Cox regression analyses of datasets GSE16091 and TARGET_OS. In all, 273 and 3450 genes were identified to be prognostically relevant, respectively (Figure 7A, 7B). Next, a LASSO regression model [16] was exploited to select the pivotal prognostic genes. With the LASSO-penalized Cox regression models, the coefficients for the top-twenty features were calculated when Log λ = -3, and the partial likelihood deviances for the selected lambda were approximately 9 and 6 as shown in Supplementary Figure 4A, respectively in the two datasets. We found that patients with high-risk scores possessed a shorter survival time compared to those with low-risk scores in all subtypes evaluated in the GSE16091 dataset (Figure 7C). Similar results were observed in the TARGET_OS dataset (Figure 7D). In addition, receiver operating characteristic (ROC) curve analysis was performed to find the diagnostic significance of these integrated genes in OS. ROC curves of in GSE16091 and TARGET_OS were displayed in Figure 7E and 7F. For 5-year survival, the average area under the curves (AUCs) were respectively 0.884 and 0.952, which showed good sensitivity and specificity. Heatmap plotted the expression profiles of genes included in the Cox model based on the low-risk or high-risk classification (Figure 8A; Supplementary Figure 4B). These results demonstrate that these genes had the highest prognostic significances for OS.

### *PSAT1* overexpression is associated with the progression and poor prognosis of OS

To further investigate the candidate genes for the diagnostic and prognostic effects of OS, we focused on one important predicted gene *PSAT1* among the 4-gene significance obtained by Cox regression in GSE16091, an oncogene that plays a vital role in cancer progression and metastasis via the serine biosynthetic pathway [12, 17]. *PSAT1* expression was up-regulated in sarcoma bone cancer tissues showing in ONCOMINE and GENT2 database (Figure 8B, 8C). Online Kaplan-Meier survival analysis revealed that *PSAT1* expression contributed to significantly shorter survival time in contrast to that maintained in lower level (HR = 2.42, *P* = 1e-5 for overall survival; Figure 8D). Considering the similar prognostic capacity of OS, we then valuated the correlation of *PSAT1* and *HSPA5* expression profiles in OS patients from GSE16091 and TARGET_OS. Of particular relevance to survival analysis, the mRNA level of *PSAT1* was positively associated with *HSPA5* in two datasets (Figure 8E, 8F).

### Dual transcriptional regulation of *ANXA1* and *PSAT1* by *HSPA5* in U-2 OS cells

**Figure 9:**
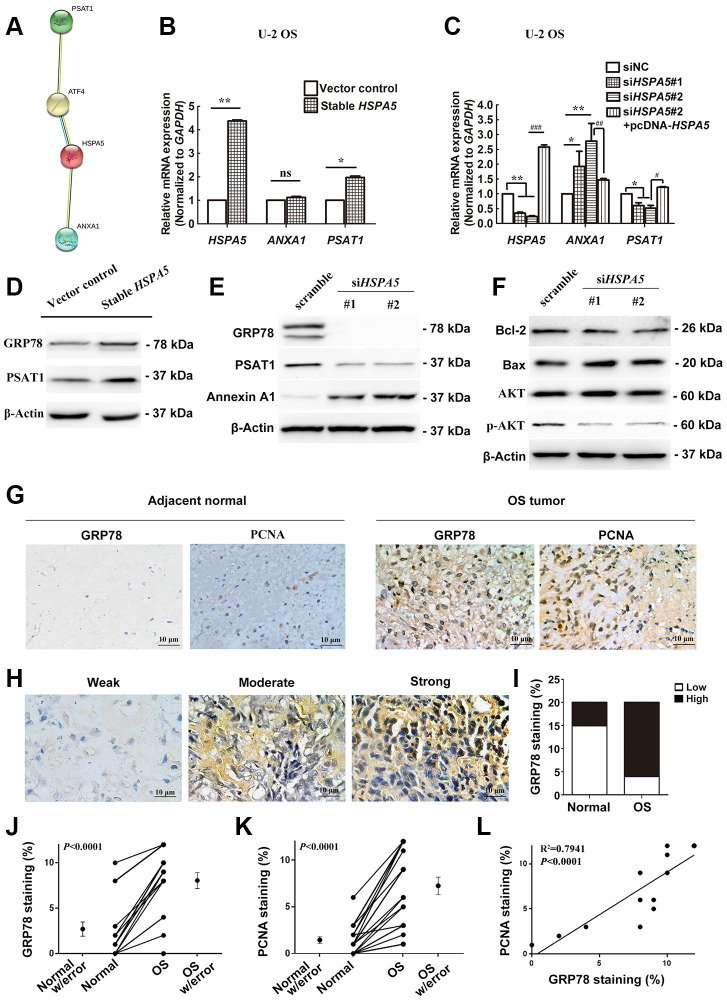
**A dual role of *HSPA5* in transcriptional mediation of *ANXA1* and *PSAT1* and its correlation with the clinicopathological features of OS.** (**A**) PPI network of proteins GRP78 (HSPA5), Annexin A1 and PSAT1. (**B**) The gene regulation of *ANXA1* and *PSAT1* in stable *HSPA5*-expressing U-2 OS cells. (**C**) The expression of the indicated genes in *HSPA5*-siRNAs and *HSPA5*-rescued plasmid-transfected U-2 OS cells. (**D**) Stable *HSPA5*-expressing or the empty U-2 OS cells were subjected to immunoblotting analysis of PSAT1 and GRP78 levels. *P < 0.05 and **P < 0.01 compared to the control. (**E**, **F**) The expression levels of (**E**) Annexin A1 and PSAT1 and (**F**) molecules involved in AKT pathway and apoptotic pathway in *HSPA5* siRNAs-transfected U-2 OS cells. (**G**) Expression of GRP78 and PCNA were detected by immunohistochemical staining in indicated adjacent normal tissues and tumors from OS patients. (**H**) Images of immunohistochemical staining for protein GRP78 in OS tissues of representative patients. (**I**) GRP78 expression increased progressively with aggressive progression of OS tissues with *P*-value less than 0.05. (**J**, **K**) IHC staining quantification of GRP78 and PCNA in the matched OS and adjacent normal tissues (n = 11 and 15, respectively). (**L**) The correlation of GRP78 and PCNA staining in OS tissues (n = 12). All data are shown with mean ± SD.*or ^#^*P* < 0.05, **or ^##^*P* < 0.01 and ***or ^###^*P* < 0.001 in comparison with the indicated group; ns, no significant difference.

Our findings suggested an interplay between *ANXA1*, *PSAT1* and *HSPA5*. STRING PPI analysis confirmed our hypothesis that GRP78 protein enabled to interacted with Annexin A1 or PSAT1 protein in a directly and indirectly manner, respectively (Figure 9A). In transcriptional level, we investigated *in-vitro* changes of the mRNA levels of *ANXA1* and *PSAT1* in stable *HSPA5*-expressing U-2 OS cells. The real-time PCR analysis showed the upregulation of *PSAT1* mRNA level (Figure 9B). Similar result was observed in the transiently *HSPA5-*transfected cells (Supplementary Figure 5A). In turn, PSAT1 loss was detected when *HSPA5* was compromised. However, *HSPA5* inhibition dramatically increased *ANXA1* transcriptional level while overexpression of which had no significant influence on *ANXA1* (Figure 9C). To exclude the possible off-target for RNAi knockdown, we performed the rescue experiment where *PSAT1* was downregulated while *ANXA1* was increased in si*HSPA5*#2-treated U-2 OS cells when reconstruction of *HSPA5* expression (Figure 9C). This suggests *HSPA5* enabled to directly regulate the transcriptional level of *PSAT1* and *ANXA1*. The present results indicate therapeutic intervention of *HSPA5* can high-efficiently block the progression of OS through dual regulation of the key diagnostic and prognostic candidates *ANXA1* and *PSAT1*. Similar to the transcriptional control, overexpression of GRP78 potentiated basic PSAT1 expression compared with the control (Figure 9D and Supplementary Figure 5B); GRP78 knockdown vice versa concurrently suppressed PSAT1 expression and upregulated Annexin A1 (Figure 9E). In addition, mitochondria play a central role in malignant tumor progression and BCL-2 family proteins were reported to be involved in mitochondria-related cell death [18]. We found the level of pro-survival protein Bcl-2 was significantly decreased, and the upregulation of proapoptotic protein Bax was observed when GRP78 was comprised. The activity of AKT signal was also impeded under such condition (Figure 9F).

### High expression of GRP78 is linked to the clinicopathological features of OS

To further illustrate the interplay between OS progression and GRP78 expression, we performed immunohistochemical analysis in bone specimens of paired tumor and adjacent normal tissues (Figure 9G, 9H). Results showed that GRP78 was overexpressed remarkably in malignant bone tissues (Figure 9G, 9I, 9J; *P* < 0.05). Elevated expression of GRP78 was observed in 16 of 20 (80.0%) OS tissues and 5 of 20 (25.0%) adjacent normal tissues (Table 1). These findings indicated that GRP78 expression was upregulated in OS tissues. The clinical correlation of GRP78 expression with clinicopathological parameters in OS further suggested that GRP78 overexpression was strongly linked to the clinical stage of OS (*P* < 0.001; Table 1). In contrast, there were no significant correlations between GRP78 expression and patient’s age, gender, tumor size, degree of differentiation, anatomic location, serum levels of lactate dehydrogenase and alkaline phosphatase or distant metastasis in OS. In addition, the expression of the cell proliferation-associated protein PCNA in OS tissues was also investigated by immunohistochemistry. In agreement with the above findings, higher levels of GRP78 protein were observed in OS tumor tissues than those in adjacent normal tissues, and there were much more PCNA-positive cells in GRP78-overexpressing tumors (Figure 9G, 9K, 9L; R^2^ =0.7941, *P* < 0.0001). Therefore, in future discovering novel drugs specifically targeting *HSPA5* may provide a potential therapeutic strategy for OS treatment.

**Table 1:** The relationship between GRP78 and clinicopathological features of osteosarcoma.

**Clinicopathological features**	**No. of cases**	**GRP78 expression**		***P*-value**
		**Low (%)**	**High (%)**	
**Tissue**				0.0005
para-carcinoma tissue	20	15 (75.0)	5 (25.0)	
Osteosarcoma tissue	20	4 (20.0)	16 (80.0)	
**Age**				0.4936
≥55	12	3 (25.0)	9 (75.0)	
<55	8	1 (12.5)	7 (87.5)	
**Gender**				0.8222
Female	11	2 (18.2)	9 (81.8)	
Male	9	2 (22.2)	7 (77.8)	
**Tumor size**				0.1432
<8	6	0 (0.0)	6 (100.0)	
≥ 8	14	4 (28.6)	10 (71.4)	
**Clinical stage**				0.0431
IIA	9	0 (0.0)	9 (100.0)	
IIB/III	11	4 (36.4)	7 (63.6)	
**Degree of differentiation**				0.608
Well and moderately	1	0 (0.0)	1 (100.0)	
Poorly	19	4 (21.1)	15 (78.9)	
**Anatomic location**				0.2636
Tibia/femur	16	4 (25.0)	12 (75.0)	
Elsewhere	4	0 (0.0)	4 (100.0)	
**Serum level of lactate dehydrogenase**				0.8073
Elevated	14	3 (21.4)	11 (78.6)	
Normal	6	1 (16.7)	5 (83.3)	
**Serum level of alkaline phosphatase**				0.4819
Elevated	13	2 (15.4)	11 (84.6)	
Normal	7	2 (28.6)	5 (71.4)	
**Distant metastasis**				0.1967
Absent	15	4 (26.7)	11 (73.3)	
Present	5	0 (0.0)	5 (100.0)	

## DISCUSSION

Osteosarcoma (OS) is one of the most common aggressive bone tumor, which is currently treated with systemic chemotherapy in combination with the surgical removal of the tumor. A major, as yet unresolved, problem is the poor prognosis characterized by chemoresistance, relapse and metastasis [1, 19]. Gene signatures identification is deemed to be vital for a better understanding of the molecular basis of OS progression, also for new targets discovery [20, 21]. At the molecular level, considerable potential biomarkers have been reported to be associated with OS prognosis [22]. The gene multiple durgresistant-1 (*MDR1*)-encoded P-glycoprotein (P-gp) has been found to be indicative of clinical OS outcomes. High levels of P-gp in OS are associated with an evident decrease in the probability of remaining progression-free after diagnosis [23]. Moreover, the co-expression of mutant p53 protein and P-pg could significantly reduce the survival time of patients [24]. Synchronous overexpression of CXCR4 and MMP9, thought to be very likely a valuable independent predictor of lung metastasis and survival in OS patients [25]. Thus, the combination of different markers for diagnosis or prognosis evaluation appears promising.

In this study, we focused on the potential oncogenes and tumor suppressors with prognostic values of OS. DEGs between the OS tumor and normal tissues across two GEO datasets were statistically analyzed. Hierarchical clustering analysis of the overlapping DEGs indicated that these genes enable to reflect the difference of tumor tissues against normal tissues. The results of GO and KEGG pathway enrichment analyses paid our attention to the biologically functional term of cell adhesion molecule binding and the protein processing in endoplasmic reticulum (ER) pathway. Shared DEGs in these two processes were obtained and further PPI analysis highlighted *HSPA5*, an oncogenic marker of great concern in OS-related research up to now. Its encoding protein glucose-regulated protein 78 (GRP78), a cell-protective ER chaperone protein, is one of the most promising anticancer targets for OS [19]. ER stress plays a significant role in allowing cells to survive when met the conditions that would normally trigger cell death. Specifically, aberrant expression of GRP78, the master regulator of the unfolded protein response (UPR), has been demonstrated to induce chemoresistance and serve as an indicator for poor prognosis in patients [26]. Overexpression of GRP78 has also been considered a predictor for aggressive behavior and poor prognosis in cancer, which was possibly involved in pathogenesis, growth, invasion and metastasis [27].

To further acquire other important prognostic markers in OS, WGCNA was constructed with the shared DEGs from the GEO and TARGET databases. A magenta module was selected due to *ANXA1* included, one of the hub genes that possessed a close interaction with *HSPA5* shown in PPI network. Annexin A1, encoded by *ANXA1*, is a member of calcium/phospholipid-binding proteins family which is responsible for mediating actin dynamics and cellular proliferation. Accumulating evidence shows Annexin A1 is a candidate regulator during carcinogenesis [28]. *ANXA1* was observed overexpressing in patients with gliomas, hepatocellular carcinoma, and adenocarcinomas of pancreas and esophagus [29, 30]. However, recent studies indicate loss of function of *ANXA1* in squamous cell carcinomas of the esophagus and head and neck, as well as in adenocarcinomas of the prostate and breast [31]. *ANXA1* expression is therefore varied depending on tumor type. Given expressing very little in the proliferating basal OS cells [32], loss of *ANXA1* is likely to be a poor prognostic factor in OS. Before functional and pathway enrichment analyses, we found a strong relevance of the magenta module containing *ANXA1* with the survival rate of OS patients. Moreover, the biological functions of this module were also enriched in the cell adhesion molecule binding process. Contrary to the role of *HSPA5*, *ANXA1* loss conferred an independent negative prognostic impact in OS. Of note, *HSPA5* knockdown significantly increased the level of *ANXA1*; however, the expression of *ANXA1* was positively correlated with *HSPA5* in OS clinical samples. Given *ANXA1* plays a role as a homeostatic protein that regulated essential transcription factors [28], and the well-known interplay between *ATF4* and *HSPA5* [9], we found *ANXA1* expression resulted in the upregulation of both *ATF4* and *HSPA5*. Silencing of *ATF4*, however, blocked the increase of *HSPA5* (Supplementary Figure 5C, 5D). This discrepancy therefore may be explained by the negative feedback loop between *ANXA1*-induced *ATF4* and its target *HSPA5*.

Following LASSO regression model identified two groups of genes to predict the survival of OS patients based on the expression profile of genes and clinical traits from GEO and TARGET databases, respectively. ROC curve analysis showed that four genes of significance (*PSAT1*, *HSD17B6*, *MYO6* and *EYA4*) appear to be potential molecular biomarkers for the prognosis of OS with relatively high specificity and sensitivity. As the top gene of such risk assessment model, *PSAT1* is deemed as a favorable prognostic marker in cancer. Increased *PSAT1* expression was significantly associated with stage of disease and metastasis in human esophageal squamous cell carcinoma (ESCC) [32]. With bioinformatics analyses and experiments, overexpression of *PSAT1* gene in nasopharyngeal carcinoma (NPC) [33], ovarian cancer [34], gliomas [35], colorectal carcinoma (CRC) [36] and non-small cell lung cancer (NSCLC) [37], was also identified as a potential diagnostic and prognostic biomarker for clinical outcomes prediction. Correlation analysis of OS clinical specimens suggested a positive relationship between *PSAT1* and *HSPA5*. It was the first evidence showing *PSAT1* might be as important as *HSPA5* to predict how poor the prognosis is for patients with OS in independent cohorts.

Taken together, this study has identified, for the first time, the comprehensive expression profile of genes in OS. A better understanding of prognostic biomarkers *HSPA5*, *ANXA1* and *PSAT1* through the network prediction and bioinformatics functional analysis provided a biological basis for the study of the progression of OS. Notably, targeting *HSPA5* alone not only significantly upregulated the level of *ANXA1* but repressed *PSAT1* expression. This bidirectionally regulation reveals that *HSPA5* may play crucial roles in OS and thus serves as a core biomarker for OS prognosis and individual-based treatment.

## MATERIALS AND METHODS

### Differential expression analysis

Three datasets of GSE16087, GSE16088 and GSE16091 [38] from the Gene Expression Omnibus (GEO) database were selected to analyze the differentially expressed genes (DEGs). The datasets for GSE16087 and GSE16088 respectively include 10 canine model of OS samples and 14 human OS samples, and also 6 normal tissue samples. GSE16091 consists of 34 human OS samples. The original. CEL files were downloaded and then were performed quality control and normalization for the matrix data of each GEO dataset as previously described [39]. DEGs of GSE16087 and GSE16088 or the overlap region were screened when |log2FoldChange (FC)| > 1 and adjusted *P*-value < 0.05. Clustering analyses were performed to show expression patterns of the DEGs in tumor and normal tissues.

### Biological function and pathway enrichment analyses

To investigate the molecular functions and pathways of the candidate DEGs in GSE16087 and GSE16088 datasets, GO and KEGG pathway analyses were conducted via the “Enrichr” package [40] in Python software. BiNGO [41], a Cytoscape plugin, is also used to assess overrepresentation of GO categories in biological networks with a versatile visualization environment. Samples from the two datasets were introduced to Python with “GSEApy” wrapper [42] for the gene set enrichment analysis (GSEA) with the cut-off criteria where nominal *P*-value < 0.05 and false discovery rate (FDR) < 0.01.

### Co-expression network construction

TARGET_OS dataset including 83 OS patients with survival data was chosen for the construction of weighted gene co-expression network analysis (WGCNA). Gene expression profiles in TARGET_OS was constructed and the shared genes with DEGs in GSE16088 were used as input data set using “WGCNA”package in R/Bioconductor software [13]. Details were described in the previous study [39]. Afterwards, we analyzed the correlation of module eigengenes with clinical traits of OS. The magenta module including *ANXA1* was selected for further analysis. Gene significance and module membership were gained to identify the hub genes associated with clinical phenotypes of OS.

### Identification of the risk signature

Univariate Cox regression was conducted to evaluate the gene expression levels in order to identify genes significantly associated with the prognosis of OS patients in GSE16091 and TARFET_OS datasets, the cut-off of statistical significance was set as *P* < 0.05. The LASSO method was then performed to select prognostic genes [43]. For prognostic model construction, stepwise multivariate Cox regression analysis was next performed on the basis of the Akaike information criterion [44]. The Kaplan Meier survival analysis was used to estimate the patient survival rates with high and low risk scores. ROC curve was used to assess the performance of the nomogram, which was generated based on the established prognostic model to predict the individual's 5-year overall survival. AUC values were calculated to designate the ROC effect.

### Cell culture

U-2 OS cell line obtained from Shanghai Advanced Research Institute, Chinese Academy of Sciences were cultured in RPMI 1640 media (Gibco, 11875093) supplemented with 10% fetal bovine serum (Gibco, 26140079) and 100 U/mL penicillin/streptomycin (Gibco, 15240112) at 37° C in a 5% CO_2_ humidified atmosphere. Stable *HSPA5*-overexpressing U-2 OS cell lines were generated by the infection of *HSPA5* EF1a-GFP/puro lentiviral vector (GenePharma, China). The transduction was carried out with the optimal multiplicity of infection (MOI). Cell colonies were selected with the complete media containing puromycin (2 μg/ml).

### Clinical specimens and immunohistochemistry

In total, 20 pairs of OS and adjacent normal tissues were obtained from the following three hospitals in Hunan, China: The Second Affiliated Hospital of Nanhua University (5 pairs), Shaoyang Central Hospital (9 pairs) and Hunan Provincial People’s Hospital (6 pairs). Clinicopathological characteristics of these patients were summarized in Table 1. Immunohistochemistry was performed according to standard protocols. Image-Pro Plus 6.0 Software was used to calculate the expression of proteins. The score criteria for the percentage of GRP78-positive tumor cells and its staining intensity refers to the previous study [45].

### Western blot analysis

Preparation of whole-cell protein lysates and the procedures for the western blot as previously described [46]. Primary antibodies against GRP78 (3177), Annexin A1(32934), Bax (5023), Bcl2 (4223), *β*-Actin (3700), AKT(9272), and p-AKT(Ser473) (4060) involved in immunoblot assays were purchased from Cell Signaling Technology (Danvers, MA, USA). Anti-PSAT1 antibody (96136) were purchased from Abcam Inc. (Cambridge, MA, USA).

### RNA extraction and qRT-PCR

Total RNA was extracted as previously described [9]. TaqMan polymerase with SYBR Green fluorescence (Nippon Gene, Japan) was applied to conduct quantitative real-time polymerase chain reaction (qRT-PCR) [8]. Analysis was performed using specific primers (*HSPA5*: sense 5’-ATCTGAGCTGGCTCCTAGAGT-3’ and antisense 5’- GCACATCTAGATCCCCGCATT-3’; *ANXA1*: sense 5’-TGCAAGAAGGTAGAGATAAAGACA-3’ and antisense 5’-GGGACCACCTTTGGATGACT-3’; *PSAT1*: sense 5’-GTCCAGTGGAGCCCCAAAAT-3’ and antisense 5’-CCCACAGACCTATGCCCTTT-3’; *GAPDH*: sense 5’-GAAAGCCTGCCGGTGACTAA-3’ and antisense 5’-AGGAAAAGCATCACCCGGAG-3’).

### siRNA and plasmid transfections

The *HSPA5* Human siRNA Oligo Duplex (SR320562), *ATF4* Human siRNA Oligo Duplex (SR319410), and the universal scrambled negative control siRNA duplex (SR30004) were purchased from OriGene Technologies Inc(Rockville, MD). The plasmid pcDNA3.1 (+)-*HSPA5* was a gift from Richard C. Austin (Addgene plasmid # 32701). The plasmid pcDNA3.1 (+)-*ANXA1* was synthesized from General Biosystems (Anhui, China). All constructs were verified by sequencing. siRNA or plasmid transfections were performed using the Lipofectamine 3000 (Invitrogen Life Technologies, USA, L3000015) according to the manufacturer’s protocol. The efficiency of knockdown and overexpression were assessed using qPCR and western blot analyses.

### Statistical analysis

All experiments were carried out with three biological replicates in a parallel manner, and results were expressed as the mean ± SD. GraphPad Prism 6 software (San Diego, CA, USA) was used to determine the differences between the groups. The protein levels of GRP78 and PCNA in paired nontumor and tumor tissues were compared by the paired Student t test. One-way ANOVA test or two-tailed Student's t-test was performed for data comparisons. *P* < 0.05 was considered statistically significant. The significance of survival time differences was analyzed by Kaplan-Meier curves using R programming of “survival” and “survminer” package. The median expression of *HSPA5* and *ANXA1* was chosen as the cut-off value.

## Supplementary Material

Supplementary Figures
