# Supplementary material for: Therapeutic potential of targeting HSPA5 through dual regulation of two candidate prognostic biomarkers ANXA1 and PSAT1 in osteosarcoma
Source: Aging (Albany NY). 2020 Dec 3;13(1):1212–35. doi: 10.18632/aging.202258 (PMC7835002; doi:10.18632/aging.202258)
Supplement: Supplementary Figures [file aging-13-202258-s001.pdf]

SUPPLEMENTARY FIGURES

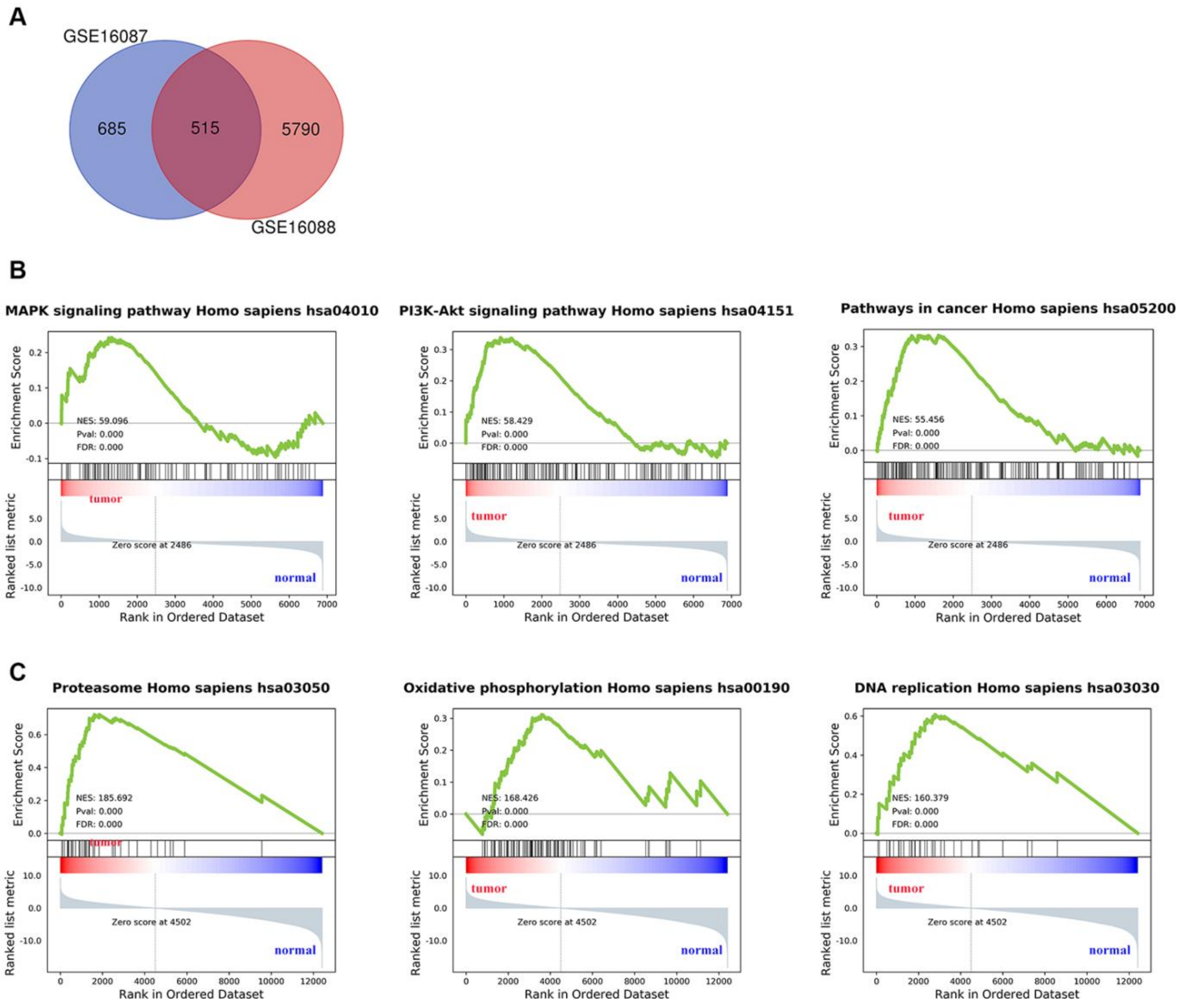

Supplementary Figure 1. Significantly enriched pathways of DEGs in OS. (**A**) Common DEGs among GSE16087 and GSE16088 datasets. (**B**, **C**) GSEA of the core gene sets from KEGG pathway among DEGs from GSE16087 and GSE16088, respectively.

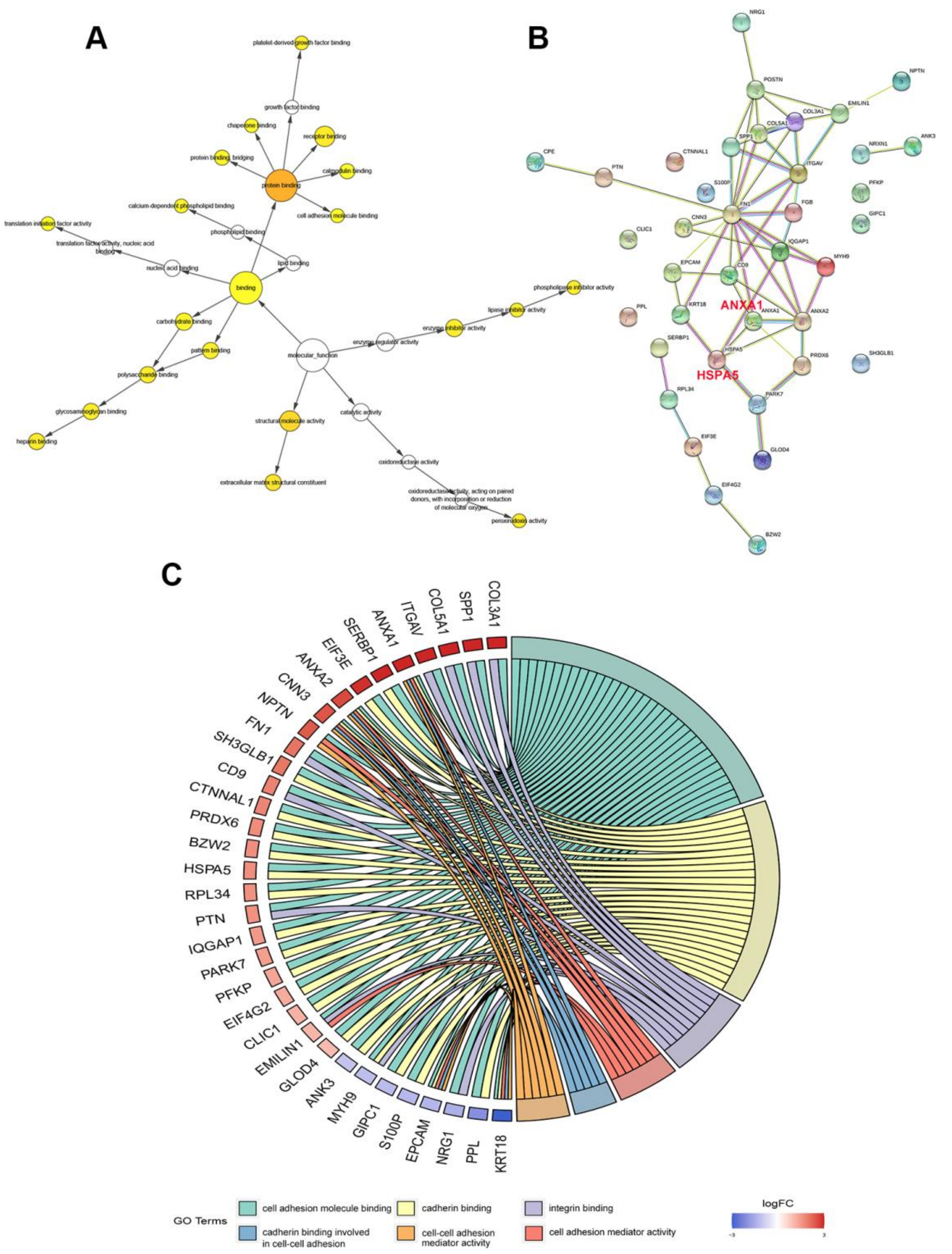

Supplementary Figure 2. Gene Ontology enrichment and protein-protein interaction analyses among the set of genes of interest in OS. (**A**) Biological network GO analysis of shared DEGs in GSE16087 and GSE16088 datasets regarding cell adhesion molecule binding process. (**B**) PPI network of identified DEGs in the two datasets in terms of cell adhesion molecule binding (**C**) Gene ontology analysis of DEGs overlapping in cell adhesion molecule binding process from GSE16087 and GSE16088 datasets.

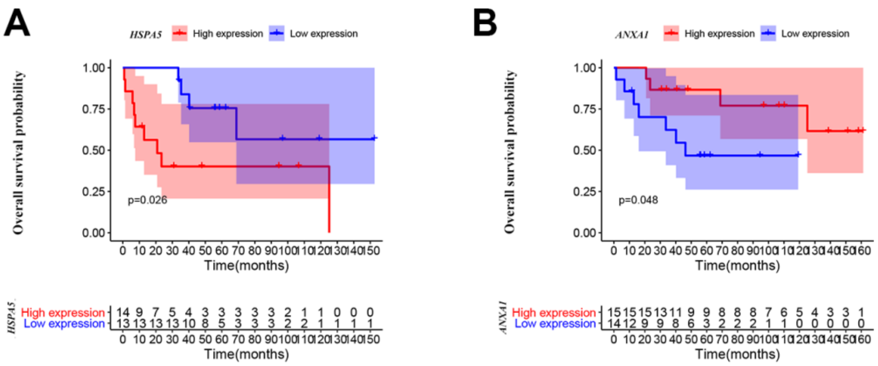

Supplementary Figure 3. High *ANXA1* expression and low *HSPA5* expression are involved in good outcomes of OS patients. (**A**) The correlation of *HSAP5* expression with the overall survival (69 cases) of OS patients shown in Kaplan-Meier curves from GSE16091 dataset. (**B**) Kaplan-Meier analysis of overall survival (in 81 cases) was conducted to show OS patients with lower *ANXA1* mRNA level live shorter from GSE16091 dataset.

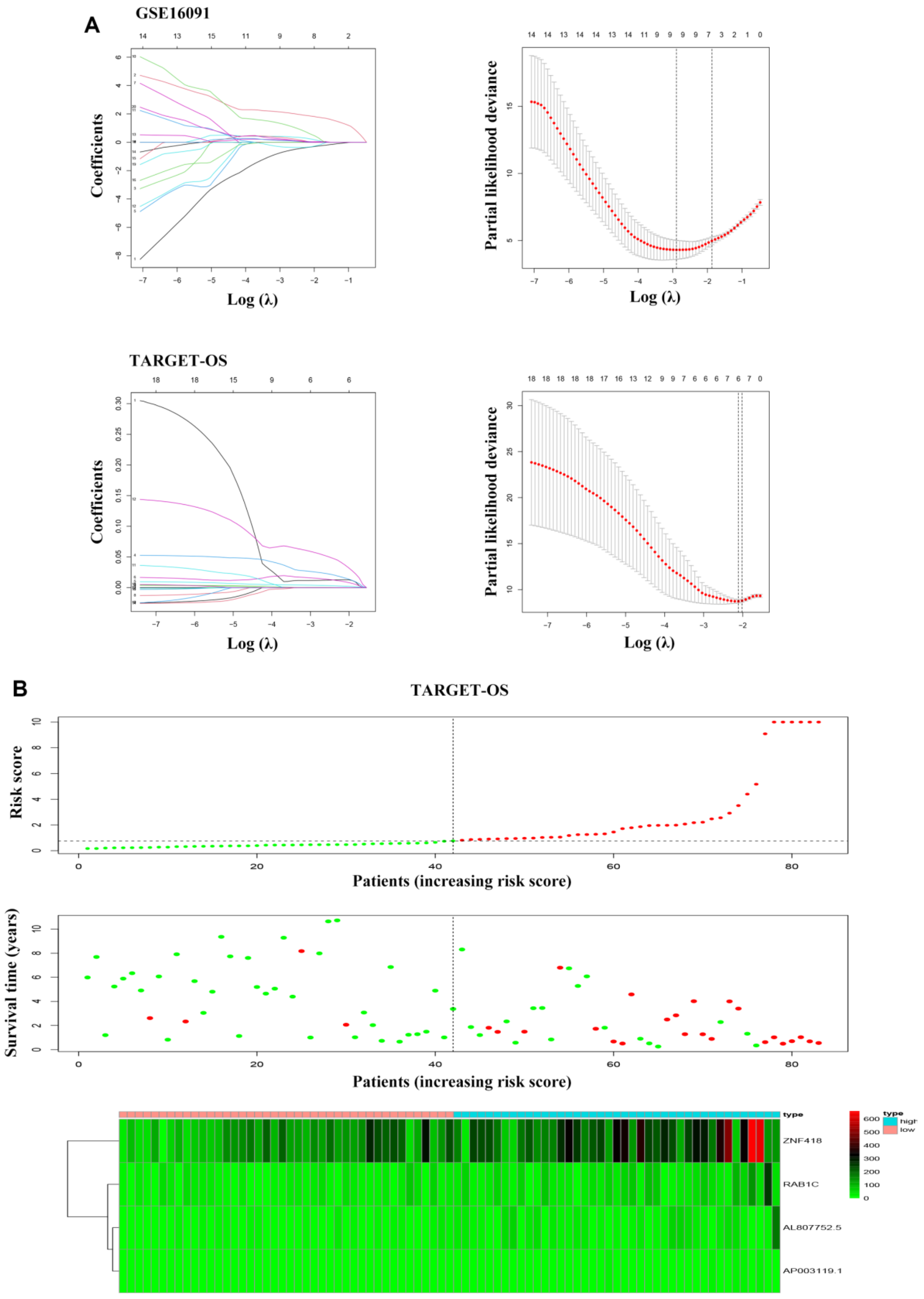

Supplementary Figure 4. LASSO regression model for prognosis predictors selection in OS. (**A**) LASSO coefficient analysis of 20 selected prognosis-related genes from GSE16091 and TARGET_OS datasets, respectively. Curves represent the coefficients. The regularization penalty parameters are shown in the x-axis. (**B**) The distribution of risk scores is shown for the cohorts from TARGET_OS (upper panel). The survival are shown in green, while the dead are shown in red. And the heatmap of expression profiles of prognostic gene signature.

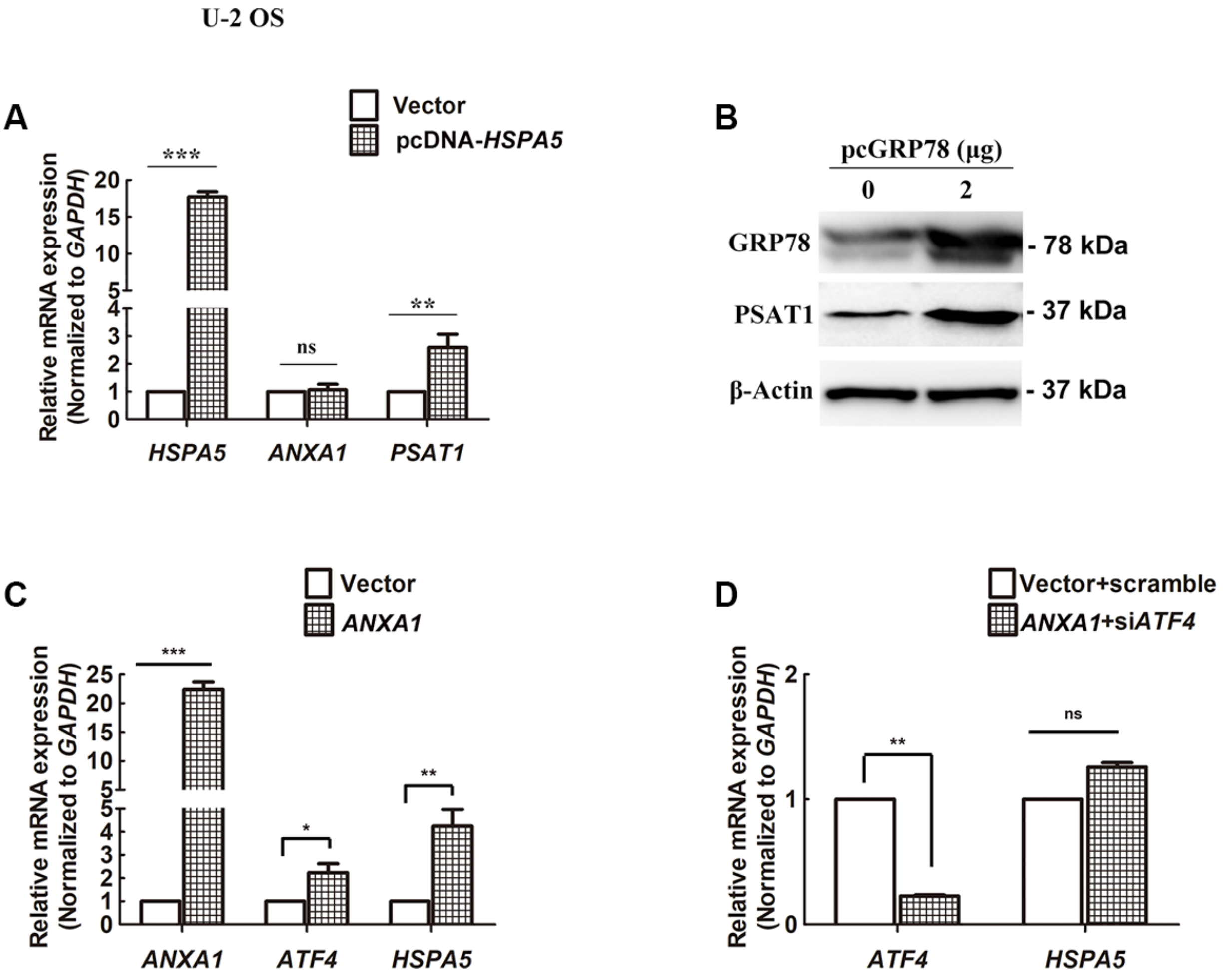

Supplementary Figure 5. The effect of *HSPA5* on the regulation of *PSAT1* and *ANXA1* in U-2 OS. (**A**, **B**) The gene regulation of *PSAT1* and *ANXA1* by *HSPA5*. QRT-PCR and western blot analyses of the indicated genes or proteins in GRP78 expression plasmid-transfected U-2 OS cells. *$P < 0.05$ and **$P < 0.01$ compared to the control. (**C**) *ATF4* and *HSPA5* mRNA levels in U-2 OS cells when subjected to *ANXA1* overexpression plasmid or the empty pcDNA3.1(+) construct. (**D**) The mRNA level of *HSPA5* in *ANXA1* overexpressing U-2 OS cells when subjected to *ATF4*-targeted siRNA or the scramble control.
